# A three-lncRNA expression signature predicts survival in head and neck squamous cell carcinoma (HNSCC)

**DOI:** 10.1042/BSR20181528

**Published:** 2018-11-21

**Authors:** Peng Wang, Meng Jin, Chuan-hui Sun, Like Yang, Yu-shan Li, Xin Wang, Ya-nan Sun, Lin-li Tian, Ming Liu

**Affiliations:** Department of Otorhinolaryngology, Head and Neck Surgery, the Second Affiliated Hospital of Harbin Medical University, Harbin, 150081, P.R. China

**Keywords:** Head and neck squamous cell carcinoma, Long non-coding RNAs, prognosis, Signature, Survival

## Abstract

Increasing evidence has shown that long non-coding RNAs (lncRNAs) have important biological functions and can be used as a prognostic biomarker in human cancers. However, investigation of the prognostic value of lncRNAs in head and neck squamous cell carcinoma (HNSCC) is in infancy. In the present study, we analyzed the lncRNA expression data in a large number of HNSCC patients (*n*=425) derived from The Cancer Genome Atlas (TCGA) to identify an lncRNA expression signature for improving the prognosis of HNSCC. Three lncRNAs are identified to be significantly associated with survival in the training dataset using Cox regression analysis. Three lncRNAs were integrated to construct an lncRNA expression signature that could stratify patients of training dataset into the high-risk group and low-risk group with significantly different survival time (median survival 1.85 years vs. 5.48 years; *P*=0.0018, log-rank test). The prognostic value of this three-lncRNA signature was confirmed in the testing and entire datasets, respectively. Further analysis revealed that the prognostic power of three-lncRNA signature was independent of clinical features by multivariate Cox regression and stratified analysis. These three lncRNAs were significantly associated with known genetic and epigenetic events by means of functional enrichment analysis. Therefore, our results indicated that the three-lncRNA expression signature can predict HNSCC patients’ survival.

## Introduction

It is widely considered that head and neck squamous cell carcinoma (HNSCC) is one of the most prevalent and fatal cancers. Despite more efforts have been taken in the prevention and treatment of HNSCC, people who get HNSCC is still increasing in recent years and the outcome remains unsatisfactory with 5-year survival rates are less than 50% [1,2]. Smoking, alcohol and human papillomavirus (HPV) infections have been associated with HNSCC dramatically [3,4]. Due to the molecular heterogeneity and diverse etiology of head and neck tumors, it is of significance to identify novel molecular biomarkers to improve the outcome of HNSCC patients.

RNAs can be separated into coding RNAs and non-coding RNAs (ncRNAs) based on the protein-coding capacity. Long non-coding RNAs (lncRNAs) (>200 nt in length) is an important section of ncRNAs [5]. LncRNAs were thought to be transcriptional noise initially. Recent studies revealed that the lncRNAs can also serve as transcriptional, post-transcriptional and epigenetic levels [6–9]. Lots of evidence illustrated that lncRNAs have complex and wide functions in the development and progression of cancer [10–12]. For instance, *H19* is an estrogen-inducible lncRNA and it plays a key role in estrogen-induced cell proliferation in breast cancer cells [13]. The experimental study demonstrated that *H19* served as a biomarker for breast cancer diagnosis and progression. LncRNA *MEG3* as a tumor suppressor is down-regulated in cervical cancer, and *MEG3* affects cell proliferation and apoptosis by regulating *miR-21* [14]. Several expression-based lncRNA signatures have been established in a variety of tumors [15–23]. For HNSCC, biological roles of microRNA in regulating the development of head and neck cancers have been summarized in recent some reviews [24–26]. Recently, some lncRNAs have been reported to be differentially expressed in HNSCC. For example, Cao et al. [27] identified a prognostic lncRNA signature by the orthogonal partial least square discrimination analysis. However, they did not verify this prognostic lncRNA signature model in the testing dataset. Zhang et al. [28] predicted the survival of HNSCC by seven lncRNA–mRNA based risk score. Because they analyzed the dataset based on two platforms (NGS and Affymetrix HG U133 plus 2), a relatively small number of lncRNA were analyzed in their study. Therefore, the prognostic value of lncRNAs still needs to be investigated in HNSCC.

In the present study, we conducted a comprehensive study of lncRNA expression profiles across 425 HNSCC patients with clinical information to investigate the prognostic value of lncRNAs in HNSCC. Finally, we identified three lncRNAs associated with survival and constructed lncRNA expression signature based on the expression profiles of these three lncRNAs in the training dataset that was further confirmed in the testing and entire datasets.

## Materials and methods

### HNSCC datasets and patient information

The lncRNA expression profiles of HNSCC patients were downloaded from TANRIC (The Atlas of ncRNA in Cancer, http://bioinformatics.mdanderson.org/) [29]. Clinical information and features of HNSCC patients were obtained from The Cancer Genome Atlas (TCGA, https://cancergenome.nih.gov/). After removing patients without available survival information, a total of 425 HNSCC patients were used for further analysis. We annotated each of the samples according to patient barcode ID based on the available clinical information, including status, age, gender, history of neoadjuvant treatment, pathologic stage, alcohol history documented, margin status, primary lymph node presentation assessment (pN), lymph-vascular invasion present and tumor grade. Table 1 included more detailed clinical information of all 425 NSCC patients in the present study.

**Table 1:** Clinical features of all 425 HNSCC patients included in the present study

Features	Training dataset (*n*=213)	Testing dataset (*n*=212)	Entire dataset (*n*=425)
**Status**			
Alive	82(38.5)	101(47.6)	183(43.1)
Dead	131(61.5)	111(52.4)	242(56.9)
**Age**			
Young (<60)	137(64.3)	142(67.0)	279(65.6)
Old (≥60)	76(35.7)	70(33.0)	146(34.4)
**Gender**			
Female	46(21.6)	69(32.5)	115(27.0)
Male	167(78.4)	143(67.5)	310(73.0)
**History of neoadjuvant treatment**			
Neoadjuvant treatment	5(2.3)	4(1.9)	9(2.1)
Without neoadjuvant	208(97.7)	208(98.1)	416(97.9)
**Pathologic stage**			
I/II	43(20.2)	43(20.3)	86(20.2)
III/IV	142(66.7)	131(61.8)	273(64.2)
Unknown	28(13.1)	38(17.9)	66(15.6)
**Alcohol history documented**			
Alcohol	148(69.5)	141(66.5)	289(68.0)
Without alcohol	60(28.2)	69(32.5)	129(30.4)
Unknown	5(2.3)	2(1.0)	7(1.6)
**Margin status**			
Ms positive	20(9.40)	22(10.4)	42(9.9)
Ms negative	149(70.0)	142(67.0)	291(68.5)
Unknown	44(20.6)	48(22.6)	92(21.6)
**pN**			
pN positive	178(83.6)	161(76.0)	339(79.8)
pN negative	18(8.5)	27(12.7)	45(10.6)
Unknown	17(7.9)	24(11.3)	41(9.6)
**Lymphovascular invasion present**			
Lymphovascular_invasion	47(22.1)	43(20.3)	90(21.2)
Without lymphovascular invasion	98(46.0)	87(41.0)	185(43.5)
Unknown	68(31.9)	82(38.7)	150(35.3)
**Tumor grade**			
G1/G2	150(70.4)	151(71.2)	301(70.8)
G3/G4	57(26.8)	54(25.5)	111(26.1)
Unknown	6(2.8)	7(3.3)	13(3.1)

### Identification of potential prognostic lncRNA biomarkers associated with overall survival in patients with HNSCC

The 425 HNSCC patients were randomly divided into the training dataset composing of 213 patients and the testing dataset composing of 212 patients. A univariable Cox regression analysis was performed to evaluate the relationship between the continuous expression level of each lncRNA and patients’ overall survival in the training dataset. The raw *P* values were adjusted by Benjamini and Hochberg multiple comparison methods to control the false discovery rate (FDR). If the FDR was less than 0.1, the corresponding lncRNAs were statistically significant and were considered as candidate prognostic lncRNAs. Subsequently, a multivariate Cox regression was performed to calculate the contribution of each lncRNA in survival prediction. The lncRNA-based risk score model was defined as the linear combination of the expression levels of the significant lncRNAs and the multivariable Cox regression coefficient as the weight. According to the median risk score in the training dataset, the patients with HNSCC in each dataset were classified into the high-risk group and low-risk group.

### Statistical analysis

The Kaplan–Meier method was used to estimate OS time for the two groups, and the statistical significance was obtained using the two-sided log-rank test [30]. Univariate and multivariate Cox proportional hazards regression analyses were carried out with OS as the dependent variable and other individual clinical features as explanatory variables in each dataset. Hazard ratios (HR) and 95% confidence intervals (CI) were obtained [31]. The time-dependent receiver operating characteristic (ROC) curve was performed to evaluate the prognostic performance for survival prediction of the lncRNA risk score and calculate the area under the ROC curves (AUC) value [32]. All analysis was performed under the environment of the R/Bio-Conductor (version 3.4.0).

### Functional enrichment analysis

In order to investigate potential biological roles of lncRNA, the co-expressed relationship between the prognostic lncRNAs and mRNA was calculated by Pearson correlation coefficients. DAVID Bioinformatics Tool (version 6.8) was used to further functional enrichment analysis. Only GO categories of ‘Biological Process’ were considered. Functional annotation with *P*-value of <0.05 was considered be statistically significant.

## Results

### Identification of prognostic lncRNAs from training dataset

A total of 425 TCGA HNSCC patients were divided randomly into the training dataset (*n*=213) and the testing dataset (*n*=212). In order to evaluate whether the lncRNA expression was associated with patients survival, the training dataset was analyzed using univariate Cox proportional hazards regression analysis method. The experimental results were shown in Table 2. A total of three lncRNAs as prognostic lncRNAs were found to be significantly associated with patients’ overall survival (FDR adjusted *P*<0.1). These three lncRNAs were entered into the candidate pool for further selection. All of three lncRNAs (*AC002066.1, AC013652.1* and *AC016629.3*) reveal positive coefficient in univariate Cox proportional hazards regression analysis indicating that a higher level expression of these three lncRNAs was associated with shorter survival. Result demonstrated that these three lncRNAs were able to independently predict patients’ OS at a statistically significant level of 0.01.

**Table 2:** Three lncRNAs significantly associated with overall survival in HNSCC patients training set (*n*=213)

Ensembl ID	Gene symbol	Chromosomal position (GRCh38)	*P* value^1^	Hazard ratio^1^	Coefficient^1^	*β*^2^
*ENSG00000237813*	*AC002066.1*	Chromosome 7: 116,238,260-116,499,465 (-)	8.43E-05	2.207	0.792	1.504
*ENSG00000259345*	*AC013652.1*	Chromosome 15: 38,865,322-39,427,195 (-)	4.89E-05	3.748	1.321	1.337
*ENSG00000269600*	*AC016629.3*	Chromosome 19: 58,593,896-58,599,355 (-)	1.5E-04	39.778	3.683	3.714

1 Obtained from the univariable Cox’s proportional-hazards regression analysis.

2 Obtained from the multivariate Cox’s proportional-hazards regression analysis.

### The three-lncRNA signature predicts patients’ survival in the training dataset

**Figure 1:**
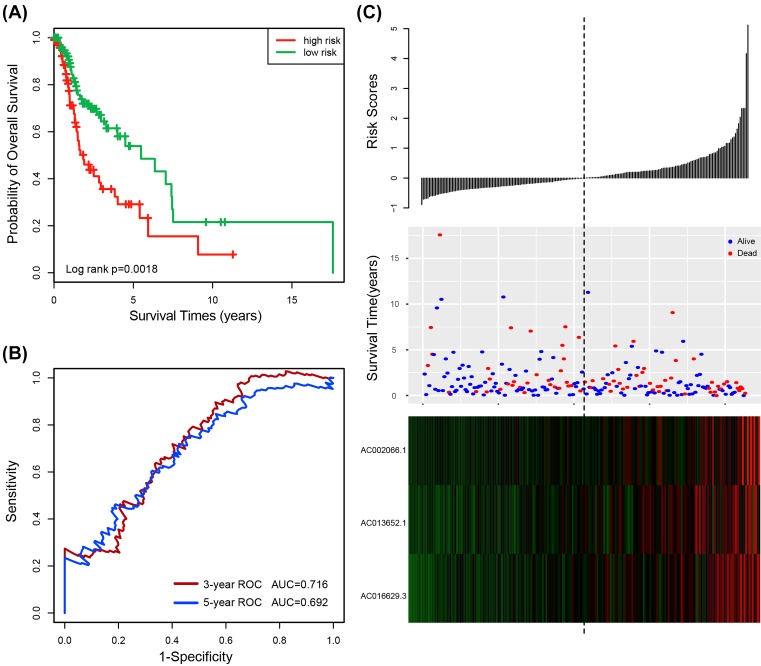
The three-lncRNA signature in the prognosis of overall survival of HNSCC patients in the training dataset (**A**) Kaplan–Meier survival estimates overall survival of HNSCC patients according to the three-lncRNA signature in the training dataset. (**B**) ROC analysis for overall survival prediction by the three-lncRNA signature within 3 and 5 years as the defining point in the training dataset. (**C**) The risk score distribution, patients’ survival status and heatmap of the three-lncRNA expression profiles in the training dataset.

In order to obtain the relative contribution of three prognostic lncRNAs to survival prediction of HNSCC, expression data of three lncRNAs were fitted by the multivariate Cox regression model in the training dataset. The contribution values (*β*) are shown in Table 2. Subsequently, a risk score model was constructed based on the expression levels of these three lncRNAs to predict the survival of HNSCC. The risk score model as follows: Risk score = (1.504 × expression value of *AC002066.1*) + (1.337 × expression value of *AC013652.1*) + (3.714 × expression value of *AC016629.3*). According to the risk score, the three-lncRNA signature risk score was computed for each patient in the training dataset. Using the median risk score as the cutoff point (cutoff = 1.010079), all patients (*n*=213) were classified into a high-risk group (*n*=107) and a low-risk group (*n*=106). The Kaplan–Meier curves illustrated that patients in the high-risk group tend to have shorter survival than those in the low-risk group (median survival 1.85 years vs. 5.48 years; *P*=0.0018, log-rank test) (Figure 1A). To determine the performance of the risk score model, the time-dependent ROC curve was analyzed. The AUC for the three-lncRNA signature risk score model at 3 and 5 years for overall survival (OS) were 0.716 and 0.692, respectively (Figure 1B). The results demonstrated the three-lncRNA signature risk score model had better performance in the training dataset. Meanwhile, the risk score was evaluated by the univariable Cox regression model analysis. The experimental results depicted that the risk score was significantly associated with patients’ OS when the risk score was analyzed as a continuous variable (Hazard ratios (HR) = 2.718, 95% confidence intervals (CI) 2.016–3.666, *P* = 5.54E-11) (Table 3). The distribution of the three-lncRNA signature risk score, the survival status and expression pattern in the training dataset were shown in Figure 1C. The expression of these lncRNAs tended to be up-regulated in patients with high-risk score (Figure 1C).

**Table 3:** Univariable and multivariable Cox regression analysis of the three-lncRNA signature and overall survival in each dataset

	Univariate analysis			Multivariate analysis		
	HR	95% CI of HR	*P*-Value	HR	95% CI of HR	*P*-value
**Training dataset (*n*=213)**						
Train score	2.718	2.016–3.666	5.54E-11	2.528	1.735–3.683	1.36E-06
Age (old/young)	1.035	0.648–1.654	0.884	0.860	0.499–1.482	0.588
Gender (male/female)	0.835	0.489–1.424	0.508	0.892	0.456–1.744	0.739
Pathologic stage (Stage III_IV/I_II)	1.558	0.864–2.808	0.140	1.536	0.751–3.145	0.240
Alcohol history documented (Yes/No)	1.027	0.629–1.676	0.915	0.992	0.548–1.796	0.979
pN (positive/negative)	0.735	0.334–1.615	0.443	0.709	0.238–2.114	0.537
**Testing dataset (*n*=212)**						
Test score	1.457	1.093–1.942	0.010	2.224	1.194–4.139	0.012
Age (old/young)	1.540	0.947–2.505	0.082	1.210	0.655–2.236	0.543
Gender (male/female)	0.783	0.480–1.277	0.327	1.178	0.592–2.346	0.640
Pathologic stage (Stage III_IV/I_II)	1.476	0.773–2.817	0.238	2.169	0.870–5.407	0.097
Alcohol history documented (Yes/No)	0.795	0.489–1.293	0.355	0.806	0.404–1.609	0.541
pN (positive/negative)	0.542	0.292–1.007	0.053	0.215	0.089–0.516	0.001
**Entire dataset (*n*=425)**						
Entire score	1.770	1.481–2.115	3.49E-10	2.096	1.574–2.790	4.02E-07
Age (old/young)	1.286	0.918–1.802	0.144	1.073	0.722–1.595	0.727
Gender (male/female)	0.837	0.588–1.192	0.325	1.124	0.704–1.796	0.624
Pathologic stage (Stage III_IV/I_II)	1.494	0.969–2.305	0.069	1.787	1.022–3.122	0.0418
Alcohol history documented (Yes/No)	0.893	0.633–1.258	0.516	0.842	0.542–1.310	0.446
pN (positive/negative)	0.640	0.396–1.034	0.068	0.356	0.182–0.695	0.002

### Validation of the three-lncRNA signature risk score model for survival prediction in the testing and entire datasets

**Figure 2:**
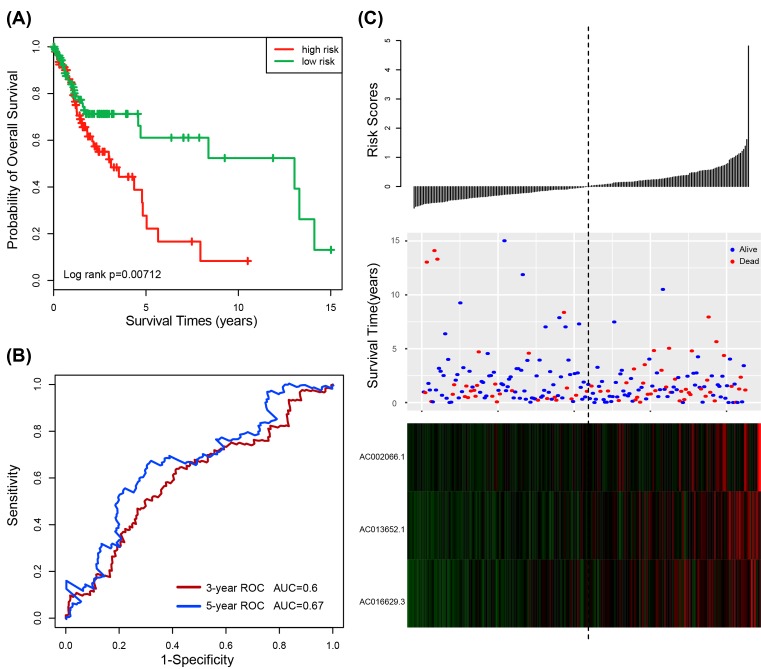
The three-lncRNA signature in the prognosis of overall survival of HNSCC patients in the testing dataset (**A**) Kaplan–Meier survival estimates overall survival of HNSCC patients according to the three-lncRNA signature in the testing dataset. (**B**) ROC analysis for overall survival prediction by the three-lncRNA signature within 3 and 5 years as the defining point in the testing dataset. (**C**) The risk score distribution, patients’ survival status and heatmap of the three-lncRNA expression profiles in the testing dataset.

In order to further validate the prognostic power of the three-lncRNA signature risk score model for patients’ OS prediction, the three-lncRNA signature risk score model was analyzed in the testing dataset (*n*=212). Using the median risk score in training dataset as the cutoff value, the patients of the testing dataset were classified into low-risk group (*n*=110) and high-risk group (*n*=102). Consistent with our findings in the training dataset, patients in the high-risk group exhibited poorer OS than those in the low-risk group (median survival 3.11 years vs. 13.04 years; *P*=0.00712, log-rank test) (Figure 2A). The AUC for the three-lncRNA signature risk score model at 3 and 5 years for OS were 0.6 and 0.67, respectively (Figure 2B). Figure 2C showed the distribution of the risk score, the survival status and the three-lncRNA expression in the testing dataset. Patients in the high-risk group tended to express risky lncRNAs at a higher level than those in the low-risk group. In the testing dataset, the significant association between risk score and OS has also been observed when the risk score was as a continuous variable in the univariate Cox regression analysis (HR = 1.457, 95% CI 1.093–1.942, *P*=0.010) (Table 3).

**Figure 3:**
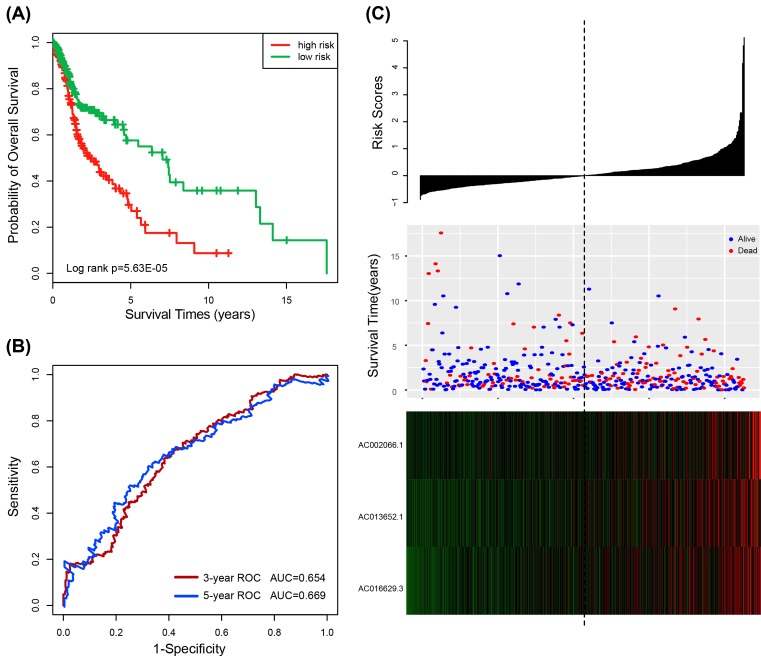
The three-lncRNA signature in the prognosis of overall survival of HNSCC patients in the entire dataset (**A**) Kaplan–Meier survival estimates overall survival of HNSCC patients according to the three-lncRNA signature in the entire dataset. (**B**) ROC analysis for overall survival prediction by the three-lncRNA signature within 3 and 5 years as the defining point in the entire dataset. (**C**) The risk score distribution, patients’ survival status and heatmap of the three-lncRNA expression profiles in the entire dataset.

The three-lncRNA signature risk score model was then tested in the entire dataset of 425 HNSCC patients. Kaplan–Meier curves showed that patients with the high-risk scores (*n*=209) had significantly shorter survival than those with the low-risk scores (*n*=216) (median survival 2.36 years vs. 7.04 years; *P* = 5.63E-05, log-rank test) (Figure 3A). Similar results were obtained by the univariable Cox regression model analysis of the three-lncRNA signature (HR = 1.770, 95% CI 1.481–2.115, *P* = 3.49E-10) (Table 3). The AUC at 3 and 5 years for overall survival was 0.654 and 0.669, respectively (Figure 3B). The distribution of risk score, the survival status and lncRNA expression of HNSCC in the entire datasets also yielded similar results (Figure 3C).

### Independence of the three-lncRNA signature risk score model for survival prediction from clinical features

**Figure 4:**
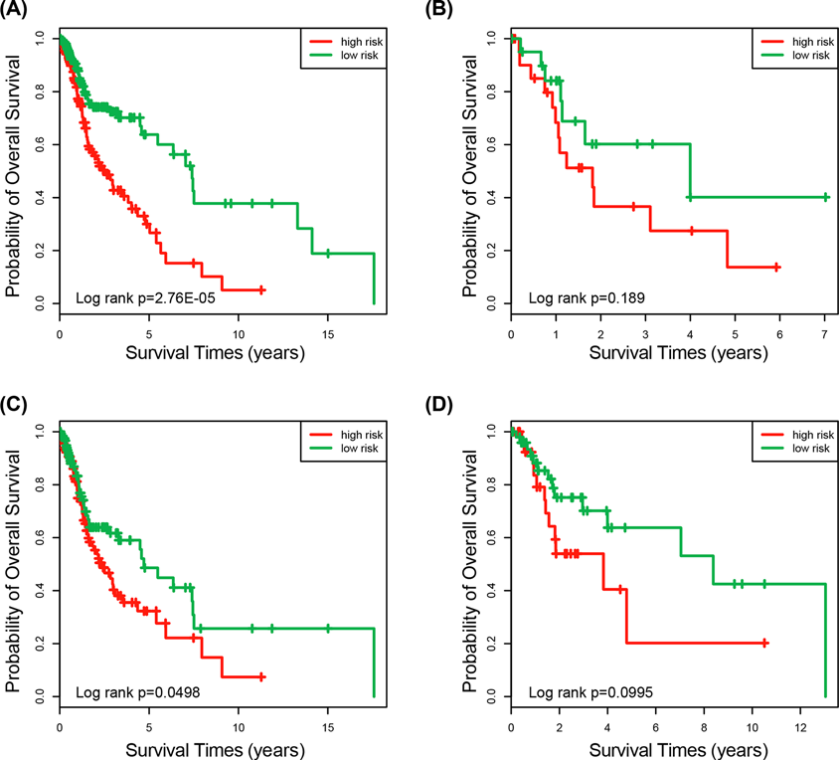
Survival analysis of all patients with pN and pathologic stage information (**A**) Kaplan–Meier curves for patients with positive of pN (*n*=339). (**B**) Kaplan–Meier curves for patients with negative pN (*n*=45). (**C**) Kaplan–Meier curves for patients with stage III/IV (*n*=273). (**D**) Kaplan–Meier curves for patients with stage I/II (*n*=86).

To determine whether the predictive ability of the three-lncRNA signature risk score model was independent of other clinical features of HNSCC patients, the multivariable Cox regression analysis was performed using lncRNA risk score and other clinical features (age, gender, pathologic stage, alcohol history documented and primary lymph node presentation assessment) in each dataset. Results demonstrated that the three-lncRNA signature was significantly associated with the survival of HNSCC patients in the training dataset (HR = 2.528, 95% CI 1.735–3.683, *P* = 1.36E-06), the testing dataset (HR = 2.224, 95% CI 1.194–4.139, *P*=0.012) and the entire dataset (HR = 2.096, 95% CI 1.574–2.790, *P* = 4.02E-07) (Table 3). Because the primary lymph node presentation assessment (pN) and pathologic stage were significant in the multivariate Cox analysis, stratification analysis was performed based on pN and pathologic stage subsequently. All patients were stratified into two subgroups with positive or negative pN. Patients with positive pN were divided into a high-risk and low-risk group based on their risk score. The Kaplan–Meier analyses demonstrated that high-risk group (*n*=171) has shorter survival than those in the low-risk group (*n*=168) (median survival 2.51 years vs. 7.41 years; *P* = 2.76E-05, log-rank test) (Figure 4A). For the patient with negative pN, low-risk group (*n*=21) also has longer survival than the high-risk group (*n*=24) (median survival 4.0 years vs.1.82 years; *P*=0.189, log-rank test) (Figure 4B). Here, the *P* that is slightly above the 0.05 significance level may be caused by too little sample size. Another clinical feature was pathologic stage that also stratified all patients into two subgroups, I/II stage patient group and III/IV stage patient group. Using the three-lncRNA signature, patients of each group could be classified into either the high-risk or low-risk group. Figure 4C showed that patients with high-risk score (*n*=145) have shorter survival than those in the low-risk group (*n*=128) in the III/IV stage patient group (median survival 2.36 years vs. 4.71 years; *P*=0.0498, log-rank test). The same results were obtained in the I/II stage patient group. The OS was significantly different in the high-risk group (*n*=34) and low-risk group (*n*=52) (mean survival 3.83 vs. 8.38 years; *P*=0.0995, log-rank test) (Figure 4D). In summary, the results of multivariate Cox regression analyses and stratification analysis illustrated that the three-lncRNA signature was independent of other clinical factors for the survival prediction of HNSCC patients.

### Functional analysis of the three prognostic lncRNAs

**Figure 5:**
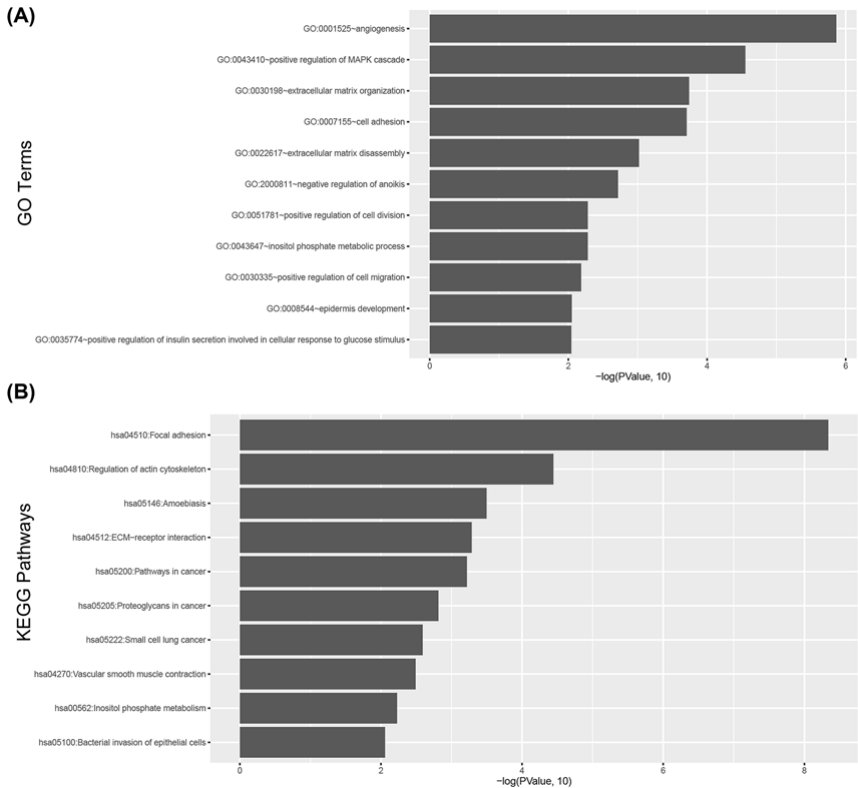
Functional enrichment results of the co-expressed protein-coding genes with prognostic lncRNAs (**A**) Significantly enriched GO terms. (**B**) Significantly enriched KEGG pathway.

To further investigate the potential biological functions of the three lncRNAs, the co-expression relationship between the expression level of the three-lncRNA and mRNA was measured. The top 5% mRNA was selected as co-expressed mRNAs with prognostic lncRNA biomarkers. The expression of 294 mRNA was correlated with the three prognostic lncRNAs. GO and KEGG pathway function enrichment analysis was employed for these co-expressed mRNA. The result revealed that 294 mRNA were significantly enriched in 11 GO terms (*P*<0.05) (Figure 5A) and 10 KEGG pathways (*P*<0.05) (Figure 5B). Some related GO terms were observed including angiogenesis, positive regulation of MAPK cascade, extracellular matrix organization, cell adhesion, extracellular matrix disassembly, negative regulation of anoikis, positive regulation of cell division, inositol phosphate metabolic process, positive regulation of cell migration, epidermis development, positive regulation of insulin secretion involved in cellular response to glucose stimulus.

## Discussion

HNSCC is one of the most commonly occurring cancers [33]. Despite considerable advances in clinical research and new therapies, the overall survival rate of HNSCC remains low. Even if the patient has the same clinical and pathological stages, their prognosis is also different. Studies have found that molecular markers can effectively distinguish between these conditions. Therefore, it is an urgent need to identify novel biomarkers to predict the patient’s prognosis. Previous some studies have illustrated that mRNA and miRNA could act as molecular markers to predict the outcome of HNSCC [34–37]. Recent studies have found that lncRNAs can also act as molecular markers in HNSCC. Through comparing 60 pairs of HNSCC tissues/non-tumor tissues samples and 7 cohorts of HNSCC cell lines, lncRNA HNSCC glycolysis-associated 1 was up-regulated in tumor tissues [38]. The results indicated that the differential expression of lncRNA was associated with the occurrence and development of HNSCC. Therefore, lncRNA can be used as a molecular marker to open a new vision for further investigation. Recently, researchers have shown that lncRNA played an important role in different cancers by analyzing expression profiles, and several lncRNA signatures in HNSCC have been identified [27,39,40]. However, the prognostic power of expression-based lncRNA signature for predicting survival in HNSCC patients still needs further investigation.

In the present study, lncRNA expression profiles of 425 HNSCC patients were obtained from TCGA except for the patient whose survival time data were missing. We identified the three-lncRNA signature by Cox regression analysis. Moreover, the regression coefficients using multivariable Cox regression analysis was obtained. Based on the risk model of the three-lncRNA signature in training dataset, the patients were classified into two groups. Patients in the high-risk group tended to have lower OS than patients in the low-risk group. Subsequently, risk score was further confirmed in the testing dataset and entire dataset. The results showed that the prognostic value of the three-lncRNA signature risk model is robust and reliable for survival prediction in HNSCC. Afterward, multivariate Cox regression analysis was performed to determine whether the predictive ability of the three-lncRNA signature was independent of other clinical features of HNSCC patients. The estimations of HR for OS were 2.528, 2.224 and 2.096 in the training dataset, the testing dataset and the entire dataset, respectively. Because pN and pathologic stage were significantly correlated with patients’ OS, the independence of the three-lncRNA signature risk model for survival prediction needs further evaluation. Stratification analysis for pN and pathologic stage was carried out. The results of stratification analysis revealed that two subgroups’ OS were significantly different in two clinical prognostic variables, individually. Studies have confirmed that *AC002066.1* has been proved to up-regulate in H2 vs. LM3 cell lines. H2 is high potential for metastasis to lymph nodes (HCCLYM-H2), and LM3 is high potential for metastasis to the lung [41]. The experimental results illustrated lncRNA expression profiles related to organ-specific metastasis in hepatocellular carcinoma. Bryzghalov et al. found that *AC016629.3* showed high expression in K562 derived from a female patient with chronic myelogenous leukemia than other non-cancer cell lines. *AC016629.3* masked miRNA target sites in seven splice forms of RPL23A [42]. In conclusion, the three-lncRNA signature risk model was an independent prognostic factor for survival prediction in HNSCC.

Thousands of lncRNAs have been discovered in humans during the past decades with the development of ncRNA prediction algorithms and software [43–46], many functions of lncRNAs are still unknown. Therefore, functional enrichment analysis was used to predict their function. The analysis revealed that the three lncRNAs mainly involved in 11 GO terms and 10 KEGG pathways. Some of the GO terms were approved to be related to cancers. For example, experiments have confirmed that angiogenesis was involved in the occurrence and development of HNSCC [47], and cell adhesion was also confirmed to be related to HNSCC [48], Nohata et al. [49] found that *CAV1* mRNA mediated tumor cell migration and invasion in HNSCC.

Three major limitations existed in the present study. The first one is the three-lncRNA signature only identified and validated in TCGA dataset. Therefore, the signature required further confirmation in large cohorts in the future studies. The other one we only used is bioinformatics method to predict the three-lncRNA signature in HNSCC, thus further experiments need to be conducted. Last, the two characteristics of smoking and radiotherapy were not considered in the present study. Our team will continue to investigate this field in the future.

Through the study of lncRNA expression profiles in HNSCC patients, a three-lncRNA signature associated with OS in HNSCC patients was identified. A three-lncRNA signature was constructed to predict the survival of HNSCC patients in the training dataset, and then, the signature was validated in the testing dataset and the entire dataset. Subsequently, multivariable Cox regression analysis of the three-lncRNA signature was investigated. The results demonstrated that the prognostic power of the three-lncRNA signature was independent of other clinical features. Therefore, the three-lncRNA signature can provide novel insights to predict HNSCC patient survival.

## Abbreviations

AUC: area under the ROC curves
FDR: false discovery rate
HNSCC: head and neck squamous cell carcinoma
HPV: human papillomavirus
ncRNA: non-coding RNA
ROC: receiver operating characteristic
TCGA: The Cancer Genome Atlas
